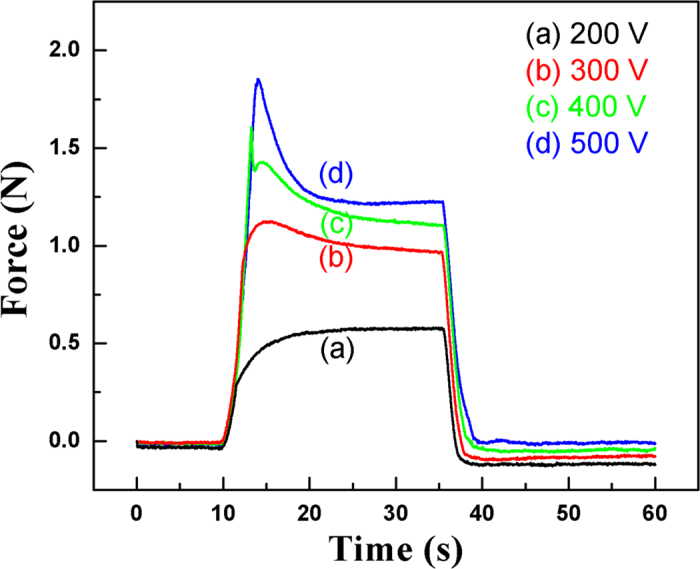# Large Displacement in Relaxor Ferroelectric Terpolymer Blend Derived Actuators Using Al Electrode for Braille Displays

**DOI:** 10.1038/srep11361

**Published:** 2015-06-16

**Authors:** S. G. Lu, X. Chen, T. Levard, P. J. Diglio, L. J. Gorny, C. D. Rahn, Q. M. Zhang

**Affiliations:** 1 Guangdong Provincial Key Laboratory of Functional Soft Condensed Matter, School of Materials and Energy, Guangdong University of Technology, Guangzhou, 510006, China; 2 School of Electromechanical Engineering, Guangdong University of Technology, Guangzhou, 510006, China; 3 Mechanical and Nuclear Engineering Department, The Pennsylvania State University, University Park, PA 16802, USA; 4 Materials Research Institute and Electrical Engineering Department, The Pennsylvania State University, University Park, PA 16802, USA

## Abstract

Poly(vinylidene fluoride) (PVDF) based polymers are attractive for applications for artificial muscles, high energy density storage devices etc. Recently these polymers have been found great potential for being used as actuators for refreshable full-page Braille displays for visually impaired people in terms of light weight, miniaturized size, and larger displacement, compared with currently used lead zirconate titanate ceramic actuators. The applied voltages of published polymer actuators, however, cannot be reduced to meet the requirements of using city power. Here, we report the polymer actuator generating quite large displacement and blocking force at a voltage close to the city power. Our embodiments also show good self-healing performance and disuse of lead-containing material, which makes the Braille device safer, more reliable and more environment-friendly.

Braille characters were coined by Louis Braille in 1821. Each Braille cell is made up of six dot positions, arranged in a rectangle containing two columns of three dots. Every dot may be raised to form sixty-four possible subsets. Currently used Braille system is composed of single line of Braille cell driven by a piezoelectric actuator for each dot such that the visually impaired people cannot read the news effectively using computer. Refreshable full-page Braille display is a novel technology aiming at significantly enhancing the information volume, from single line to multi-lines, and from only text to text including graph display^1^. Up to now, it has been pursued by many research groups in terms of various actuators, e.g., elastomers^2^, shape-memory polymers^3^, bistable electroactive polymers^4^, pneumatic^5^, ionic polymer metal composites^6^, etc. The core part of this kind of technology is the actuator. Compared with above mentioned ceramic actuators, ferroelectric polymer is one of the promising candidates^7^.

At present, the commercialized Braille actuators are lead zirconate titanate [Pb(ZrTi)O_3_ (PZT)] ceramics using their bimorph vibration modes. The advantages using PZT are their large driving force, thermal stability, and long lifetime. But obvious shortcomings are their small displacements, large cell size, heavy weight, and lead-containing. On the other side, it has been found that poly(vinylidene fluoride - trifluoroethylene – chlorofluoroethylene) (P(VDF-TrFE-CFE)) terpolymers demonstrate a transverse strain (~5%), which is much larger than that of traditional PZT ceramics (strain ~ 0.6%^8^) and even Pb(Zn_1/3_Nb_2/3_)O_3_-PbTiO_3_ (PZN-PT) (or Pb(Mg_1/3_Nb_2/3_)O_3_-PbTiO_3_ (PMN-PT)) single crystals (strain ~ 1.7%^9^). The polymers with such a large strain can be made actuators used for tactile Braille display devices. In addition, because the Young’s modulus of terpolymers is not very large, poly(vinylidene fluoride – chlorotrifluoroethylene) (P(VDF-CTFE)) copolymer with a larger Young’s modulus^10^,^11^ was incorporated into the terpolymer, increasing the Young’s modulus up to 1.5 times, while the strain maintains almost the same^12^. The introduction of P(VDF-CTFE) copolymer also makes the handling of the film easier, thus thinner films (~ 4 μm in thickness) can be more readily fabricated. Compared with other actuator materials, relaxor ferroelectric terpolymer blends P(VDF-TrFE-CFE)-P(VDF-CTFE) manifest several advantages, e.g., large displacement at room temperature, sufficiently large force, low driven electric field, light weight, self-healing, fast response speed, relatively simple manufacturing process, and environmental benignity^11^^,^^3^,^4^,^5^,^6^,^7^,^8^,^9^,^10^,^11^,^12^,^13^,^14^,^15^. The electric field driven electrostriction makes the terpolymers demonstrate large longitudinal and transverse strains, both >3% at 100 MV/m^11^. Since the electrostrictive strain is proportional to the square of electric field, even larger strain could be induced at higher electric fields.

On the actuator side, although previous actuators that were made using a spring core demonstrated large strains^7^, the pre-stress generated by the spring caused the actuators to have a shorter lifetime. In addition, the conducting polymer that we used as the electrodes before produced a thicker and nonuniform layer, which may not be suitable for the multilayer module of a Braille display^16^. Moreover, conducting polymer is also detrimental to the environment. Thus, an Al electrode is used instead in the present work.

In this work, coreless actuators were designed since spring introduces nonuniform contact with the terpolymer thin film, which will affect the actuator’s lifetime and reliability. The Al electrode is used due to the recent studies on the metal electrode deposited on an elastomeric substrate, which can withstand large strain without cracking. Another feature of Al electrode is its “self-healing”, i.e., some weak spot can be burned at a higher electric field while other part of the film can still be alive. During the fabrication, roll-to-roll stretching technique was employed to make more uniform and thinner polymer films^17^. Actuators showing displacement >0.5 mm at 300 V were produced. The blocking force generated by the actuator was measured under various electric fields.

## Results

### Electrostrictive displacement as a function of time

**Figure 1:**
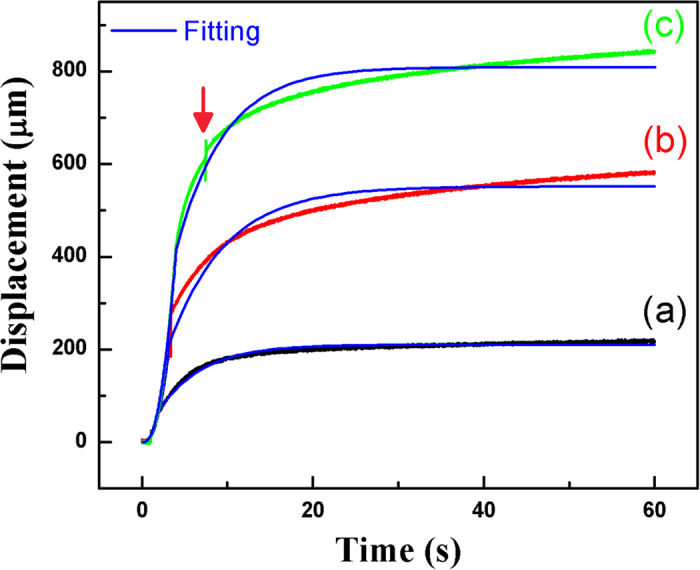
Displacement as a function of time for bilayer film with Al as electrodes at 200, 300 and 400 V. Fitting was carried out using equation (10). Arrow indicates a self-healing.

Figure 1 exhibits the typical characteristics of electrostrictive displacement as a function of time and applied voltage. Considering the charge accumulation, a charge-discharge mode with a dc voltage supply was used. In order to prevent the film from breakdown, a ramp voltage-time profile was utilized. The ramp rate was set as 100 V/s. Since the charging and creeping take a longer time, the time interval was set as 60 s for enough charging/creeping and discharging processes, respectively. The total time was set as 125 s.

The displacement obtained here can be divided into two parts, one is the fast part, coming from the electrostriction generated displacement, which is proporpotional to the P^2^. Another one is the slow part, corresponding to the creep process. For this process, traditionally the polarization creep was taken for granted for piezoelectric and/or ferroelectric ceramic or crystal materials. Since these ceramics and crystals are rigid, the polarization creep is the only reason for the creep phenomena. The polarization relaxation in a time domain makes contribution to the total strain, or displacement. The polarization relaxation with time can be expressed as^18^


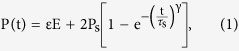


where ε is the permittivity, P_s_ the saturation polarization, τ_s_ the switching time, γ is also called the Avrami parameter that can take the values 1, 2, and 3, corresponding to the dimension parameter of nucleation growth. γ determines the shape of the switching curve. The dependence of τ_s_ on E can be written in terms of the exponential law^19^


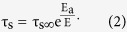


Here τ_s∞_ is a constant. It is found that the strain caused by the polarization relaxation is almost a constant in the time range 0–60 s by calculation using equation (1).

For the polymer materials or those materials with a moderate Young’s modulus, however, there is another possibility that the creep might be caused by the mechanical stress or the Maxwell stress due to the high voltage applied on the two surfaces besides the polarization creep, which is called mechanical creep. The mechanical creep generated displacement may be due to the ferroelectric and viscoelastic properties since the terpolymer blended film is a ferroelectric and also has a Young’s modulus of about 800 MPa, the Maxwell stress due to the electrostatic force plays a significant role in inducing the creep behavior.

The viscoelastic creep may be caused by the Maxwell stress σ generated by the applied electric field (σ = ε_0_εE^2^)^20^. It is interesting that this strain is also proportional to the square of electric field. Although the viscoelastic characteristics of creep process in a real material are complicated, usually the strain contains both the Maxwell strain and the Kelvin-Voigt strain in a time domain. Recently, a Standard Linear Solid model was proposed to illustrate the viscoelastic behavior of polymers, which is composed of a Maxwell model (a dashpot and a spring 1 in series corresponding to the intermolecular slippage and the bending and stretching of interatomic bonds, respectively) and an additional spring 2 (linear due to the constant rate of loading)^21^. The governing equation for the total stress can be expressed as^21^


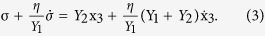


For creep behavior of the polymer blend, 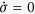, σ = σ_0_, the Maxwell stress caused by the applied electric field, and 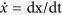, then the Standard Linear Solid model can be simplified as


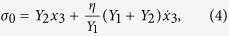


here η is the viscosity which can be approximately regarded as a constant during the creep period^21^, Y_1_ and Y_2_ are the Young’s modulis of spring 1 and spring 2, x_3_ the strain along the electric field direction, i.e., the thickness direction. The solution of equation (4) can be very easily deduced as


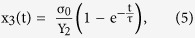


where τ = Y_2_/(η(Y_1_+Y_2_)/Y_1_), σ_0_ = εε_0_(V/l(1+x_3_))^2^, l is the thickness of the terpolymer/copolymer blended film. Usually the strain and electric displacement in thickness direction of the blended film are associated with the electric polarization and the stress in terms of the elastic and electrostrictive properties. The equation of state can be written as^22^


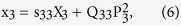


Here X_3_ is the stress, P_3_ the electric polarization, s_33_ the elastic compliance coefficient, Q_33_ the electrostrictive strain coefficient. Combining the strain caused by the Maxwell stress, the total strain can be expressed as


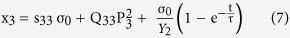


Since the strain measured for polymer blends is less than 4%, the Maxwell stress can be simplified as σ_0_ = εε_0_(V/l)^2^, and its direction is opposite to the x_3_. Then x_3_ can be written as


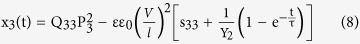


Then the axial strain x_1_ can be written as below in accordance with the definition of Poisson’s ratio,


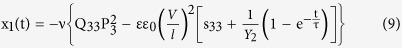


here ν is the Poisson’s ratio.

Considering the ramp rate k = (V/t) used during the strain measurement, equation (9) can be further expressed as


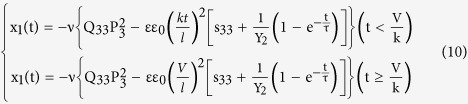


Here P_3_ is actually a function of time, and in the form of equation (1).

We consider two extreme cases, 1) when t is 0, equation (10) can be written as


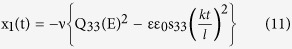


This is a parabolic function of time t. Because Q_33_ < 0, x_1_ is the summation of two terms and is proportional to t^2^.

2) when t approaches ∞, equation (10) has the form,


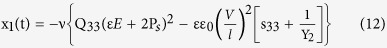


This equation is independent of the time t. It means that the strain tends to be a constant.

The displacement as a function of time and voltage for a typical actuator was fitted using equation (10). Results are presented in Fig. 1. First of all, the results are fitted well. It means that the whole relaxation consists of two processes, one is fast, and another is slow. Secondly, for the slow process, both the polarization and the Maxwell stress derived mechanical creeps make contribution to the total displacement. In fact, when γ = 1, equation (1) has the similar exponential function form with respect to time t as equation (8) except for the different time constants, indicating that two types of creeping follow the similar time dependence, only the switch times are different since the electrostrictive displacement is proportional to P^2^. They might be existed simultaneously, but it is hard to distinguish them, especially when t approaches ∞. Thirdly, the displacement goes up with time during the slow process. This is different from what the two creeps behave in the piezoelectric ceramics and metals. This slow increase of polarization versus time has been observed by Schutrumpf *et al.* in normal P(VDF-TrFE) films^18^ and Mai *et al.* in Langmuir-Blodgett (LB) films^23^. Although the observation clearly indicates the increase of polarization, one cannot exclude the impact of Maxwell stress on the polarization in terms of the decrease of thickness. Schutrumpf urges that the screening charges might be accumulated at the interface boundaries of non-ferroelectric/ferroelectric layers, charges are travelling through the non-ferroelectric portions of the film and build an interface charge layer at the phase boundaries^18^. It seems that it is hard for an electrical field as small as 60 MV/m for normal films and 43 MV/m for LB films to drive the charges travelling across the insulative non-ferroelectric phase. On the contrary, it is easier for an electric field to change the polarization as well as the strain of the copolymer film in terms of the electrostriction and Maxwell stress. Fourthly, calculation indicates that if the Young’s modulus is as low as 200 MPa, the permittivity is 40, the external electrical field is 100 MV/m, the Maxwell stress derived strain is about 1.8%, which numerically means the Maxwell stress might play a great role in affecting the strain properties of the polymer films. Moreover, very recently, it was discovered by Gruverman and Catalan *et al.* that the ferroelectric polarization can be switched by the stress gradient in the nanoscale volume of a ferroelectric film through the flexoelectricity, a coupling between the polarization and strain gradient, which may be added to illustrate the synergistic action to the viscoelastic properties of blended polymers^24^.

### Electrostrictive Force as a Function of Time

The mechanical forces produced by the actuator at various voltages are recorded as a function of time. The results for a bilayer actuator are shown in Fig. 2. One can see that the actuator may generate over 1 N at 300 V. A decay of force with time was observed during the force measurement, which could be seen as the stress relaxation in terms of Maxwell model in the polymers. It is reasonable that the boundary was fixed during the force measurement, i.e., the strain is a constant. This is the situation of Maxwell model. Thus a decay of stress versus time is expected to be observed.

The stress relaxation modulus can be given as


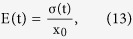


where strain x_0_ is a constant. With the increase of applied voltage, the whole stress relaxation modulus versus time curve rises up, indicating the blocking force increases due to the electrostrictive actuation and viscoelastic creep. In addition, the decay speed of blocking force increases with the applied voltage, indicating the relaxation time becomes shorter and shorter. It seems that the applied voltage may reduce the viscoelasticity such that the viscosity is reduced, then the relaxation time will be reduced as well. After that there is a slow response process, which is related to the release of blocking force, or creep behavior.

It should be noted that the mechanical behavior beyond the viscoelastic limit, i.e. the buckling of the actuator. Although the actuators are composed of several layers which look like cylinders, the response produced in the actuator does not satisfy with the cylinder model, but with the Euler’s column model instead. For the real measuring configurations of the actuators, usually one end was fixed and the other was free. Considering the compressive force applied along the axis of the actuator, the terpolymer blended films have a thickness d_0_, length L (here L > 2r_0_), width w, Poisson’s ratio ν, Young’s modulus Y, and the rolled actuator has a radius of r_0_, then the critical buckling load is^25^


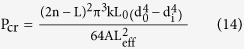


here n denotes the nth mode of buckling, representing the corresponding deflection behavior, k is the stiffness of the actuator, L_0_ is the initial length of the actuator minus the length of the steel electrodes, d_0_ is the outer diameter, d_i_ is the inner diameter, A is the cross sectional area, L_eff_ is the effective length. The cross sectional area of the actuator is calculated by multiplying the bilayer thickness by the length of the film in the rolling direction. It was found that all the buckling results are located between the 1^st^ and 2^nd^ modes^25^. Using the following data, Y = 0.8 GPa,^10^ ν = 0.392 (for PVDF^26^), d_0_ = 4 μm, w = 75 mm, then P_cr_ = 2.7 N is obtained. The critical buckling load is less than what we got before using Euler buckling model (~9 N)^16^. The main reason is that the actuator is tubular, not a rod, which will decline the critical load very much. For the experimental results (not shown here), the force generated at 500 V is less than the critical buckling load. As mentioned above, the decay is believed to come from the viscoelasticity of the polymers due to the constant strain boundary condition. In fact the actuator was still alive after the force measurement, no any buckling trace was observed on the actuator surface.

The P(VDF-TrFE-CFE)-P(VDF-CTFE) blend films were fabricated via a solution casting method with evaporated Al as electrodes and Au as edge electrode for ohmic contact. The blend films were rolled into core-free actuators for Braille display devices. The actuators may offer displacement of 0.5 mm, and force of 1.1 N at 300 V, which surpass the two critical requirements for Braille display devices. The creep phenomena were accounted for the polarization and Maxwell stress induced creeps, and fitted well with an equation deduced from equations of state and Standard Linear Solid model. The buckling was ascribed to the cylinder structure of the actuator, and a maximum buckling load of 2.7 N was estimated.

## Methods

### Materials preparation

The blend polymers are composed of a mixture of a terpolymer (P(VDF-TrFE-CFE) 64.3/27.6/8.1 mol.%) and a copolymer P(VDF-CTFE) (91/9 mol%), in which the content of copolymer is ~4 wt%.

The “green” blend film was fabricated through a solution casting method. The solvent used was 1-methyl-2-pyrrolidinone (NMP) (C_5_H_9_NO). After weighing, the mixed solution was stirred at room temperature or at an elevated temperature (40–50 °C) for 10–24 hrs. Then the solution was cast onto a quartz plate and dried at 70 °C for overnight (12 hrs or more). Then the green film was peeled off from the quartz plate in d. i. water and dried at 60–85 °C in a vacuum oven for 10–30 hrs. The next step was stretching the film by 4 to 6 times using zone-heating stretching (zone temperature 20–40 °C) or roll-to-roll stretching at the same temperature in a refrigerator environment (below 0 °C). The thickness can be adjusted by controlling the concentration of the blend solution as well as the stretching ratio. After annealing the green film at 105 °C in an oven for 5–15 hrs, the blended film with desired thickness can be obtained. The final thicknesses of the blended films are in the range of 4 to 6 μm.

The Al electrode was evaporated on both sides of the blended film. Then Au was sputtered at the edge of the Al electrode area to reduce the contact resistance, because usually the Al could be oxidized to some extent. In order to laminate the two blended layers, a three-side-electrode configuration was used (see Supporting Information). The lamination was carried out at 90-105 °C for 1 hr. Then the bilayer film was rolled into an actuator with diameter of about 2.2 mm and a length of 40 to 45 mm after annealed in a vacuum oven and cutting the two ends. The silver conductive adhesive was used to glue a stainless steel ball at the two ends to be used as contact electrodes. In order to measure the displacement of the rolled actuator, a special cell was designed in which the actuator can be stand using two magnetic discs, which are connected with the external electric field, and can reflect the laser light at the top end.

### Electrostrictive strain and blocking force measurements

The displacement was measured using a Vibrometer (Polytec OFV 3001) with a Fiber Interferometer (Polytec OFV 511). The high voltage was supplied with a Trek power supply (Model 610E). The force produced by the actuator at various voltages is recorded as a function of time using a force sensor (Load cell EBB-1, Transducer Techniques, California).

## Additional Information

**How to cite this article**: Lu, S. G. *et al.* Large Displacement in Relaxor Ferroelectric Terpolymer Blend Derived Actuators Using Al Electrode for Braille Displays. *Sci. Rep.* **5**, 11361; doi: 10.1038/srep11361 (2015).

## Supplementary Material

Supplementary Information

## Figures and Tables

**Figure 2:**