# Supplementary material for: Large Displacement in Relaxor Ferroelectric Terpolymer Blend Derived Actuators Using Al Electrode for Braille Displays
Source: Sci Rep. 2015 Jun 16;5:11361. doi: 10.1038/srep11361 (PMC4468841; doi:10.1038/srep11361)

Additional information

Large displacement in relaxor ferroelectric terpolymer blend derived actuators using Al electrode for Braille displays

S. G. Lu, X. Chen**, T. Levard, P. J. Diglio, L. J. Gorny, C. D. Rahn, and Q. M. Zhang*

Preparation of bilayer structure

The electrode configuration of 3-sided bilayer lamination is shown in Figure S1. The Al electrode was evaporated on the blend film. Then Au was sputtered at the edge of the Al electrode area to reduce the contact resistance. Since 3-sided configuration is used, only one side of the second film is electroded, the electrode of the first film will be the common electrode of the bilayer structure by thermal lamination. Dielectric measurement indicates that the total permittivity is almost the double value of the single film, which means the lamination is pretty good.

Dielectric and mechanical properties

Figure S2(a) shows the permittivity as a function of temperature for frequencies at 1, 10, and 100 kHz. These are typical dielectric characteristics of relaxor ferroelectrics, i.e. the permittivity peak shifts towards higher temperatures with increasing frequency. At the same time, peak values decline with frequency. Compared with pure terpolymers, the permittivity of blends reduces from 55 to 45 at 1 kHz and room temperature due to the incorporation of P(VDF-CTFE) copolymer, whose permittivity is 13 at 1 kHz and room temperature. Their loss tangents are almost the same at room temperature. Figure S2(b) exhibits the permittivity as a function of frequency for double-side electroded single layer and three-side electrode laminated layer. One can see that both of them show similar permittivities and loss tangents, indicating the lamination is pretty good.

It was found that the Young's modulus of the blends is about 800 MPa, larger than that of pure terpolymer (~ 500 MPa) (data not shown here)[S1]. The Young's modulus reduces monotonically with temperature. The strain maintains almost the same as pure terpolymer. These properties make the terpolymer blended film have larger mechanical energy (~ $1/2YS^2$), and further generate larger electrical energy during the electromechanical energy conversion. At the same time, it also makes the handling of films easier.

Reference

S1. L. Gorny, S. G. Lu, S. Liu and M. Lin, *IEEE Trans. Ultra. Ferro. Freq. Contol.* **2013**, *60*, 441.

Figure S1. Electrode configuration of 3-sided bilayer laminate. (a) top electrode of second layer; (b) top electrode of first layer; (c) bottom electrode of first layer.

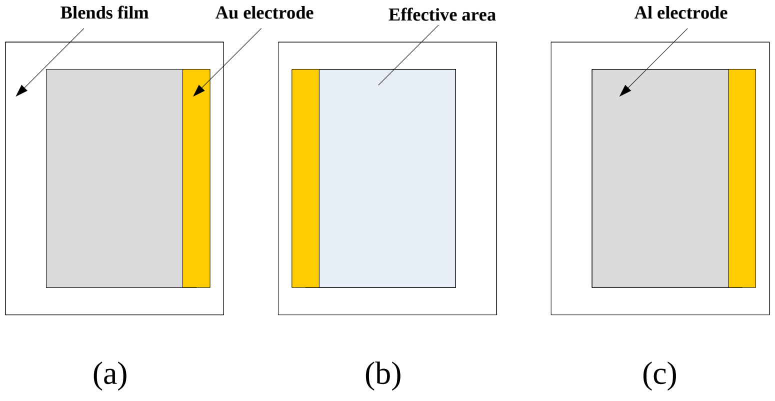

Figure S2. (a) Permittivity versus temperature for blended film. (b) Permittivity versus frequency for double-sided layer and laminated layer.

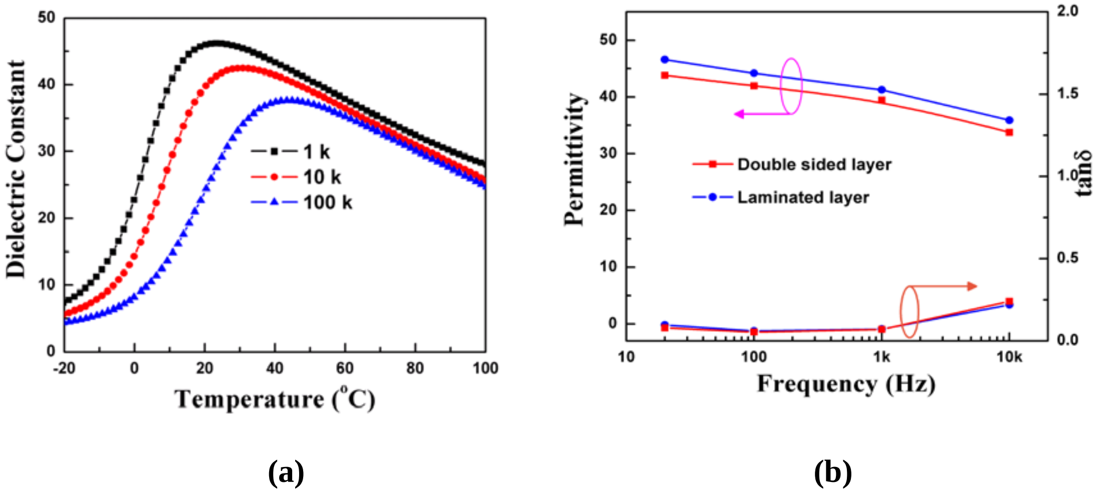

Supplement: Supplementary Information [file srep11361-s1.pdf]
